# Acceptability and feasibility of HIV recent infection surveillance by healthcare workers using a rapid test for recent infection at HIV testing sites — Malawi, 2019

**DOI:** 10.1186/s12913-022-07600-7

**Published:** 2022-03-15

**Authors:** Melissa M. Arons, Kathryn G. Curran, Malango Msukwa, Joe Theu, Gabrielle O’Malley, Alexandra Ernst, Ireen Namakhoma, George Bello, Carson Telford, Vedapuri Shanmugam, Bharat Parekh, Evelyn Kim, Trudy Dobbs, Danielle Payne, Salem Gugsa

**Affiliations:** 1 Division of Global HIV and Tuberculosis, Center for Global Health, Centers for Disease Control and Prevention, Atlanta, GA USA; 2 Department of Global Health, International Training and Education Center for Health (I-TECH), University of Washington, Lilongwe, Malawi; 3 Department of Global Health, International Training and Education Center for Health (I-TECH), University of Washington, Seattle, WA USA; 4 Global Strategic Information, Institute for Global Health Sciences, University of California, San Francisco, CA USA; 5 Department of HIV AIDS, Ministry of Health, Lilongwe, Malawi; 6 Division of Global HIV and Tuberculosis, Center for Global Health, Centers for Disease Control and Prevention, Lilongwe, Malawi

**Keywords:** HIV, Surveillance systems, Healthcare workers, Acceptability, Feasibility, Point-of-care test, recent infection surveillance, RTRIs

## Abstract

**Background:**

The Malawi Ministry of Health implemented a new surveillance activity in April 2019 to detect recent HIV infections using a rapid test for recent infection (RTRI) to identify areas of ongoing transmission and guide response activities.

**Setting:**

At 23 health facilities in Blantyre District, healthcare workers (HCWs) were trained to conduct recent infection testing. In September 2019, we conducted a cross-sectional survey at these sites to explore the acceptability and feasibility of integrating this activity into routine HIV testing services (HTS).

**Methods:**

Research assistants interviewed HCWs using a semi-structured survey. Descriptive statistics were used to summarize quantitative responses and thematic analysis was used to group open-ended text.

**Results:**

We interviewed 119 HCWs. Eighty-two percent of participants reported the RTRI was easy-to-use. HCWs perceived high client acceptability; 100% reported clients as ‘somewhat’ or ‘very accepting’. Challenges included 68% of HCWs estimating they spend ≥20 min beyond routine HTS per client for this activity and 51% performing at least two additional finger pricks to complete the testing algorithm. HCWs differed in their perceptions of whether results should be returned to clients.

**Conclusion:**

This study assessed HCW experiences using point-of-care RTRIs for HIV recent infection surveillance. Overall, HCWs perceived RTRIs to be acceptable, easy-to-use, and valuable. Though only clients with new HIV diagnoses are tested for recent infection, additional time may be substantial at high-volume health service delivery points. Providing response plans or aggregated recent infection results to HCWs and/or clients may support motivation and sustainability of this novel surveillance activity.

**Supplementary Information:**

The online version contains supplementary material available at 10.1186/s12913-022-07600-7.

## Background

As countries progress towards universal coverage for HIV diagnosis, treatment, and viral suppression, continuous and timely surveillance remains essential to ensure public health interventions are effectively reaching those at highest risk of acquiring HIV. Strategies to identify populations with elevated HIV transmission and rapidly intervene, to stop the chain of transmission, can help to reach this goal. In Malawi (adult HIV prevalence: 8.9%), an estimated 90% of people living with HIV knew their status, of which 88% were on treatment, and of which 92% were virally suppressed in 2019 [1]. According to a 2015-16 population-based survey in Malawi, Blantyre had the highest adult HIV prevalence at 17.7%, making it a priority district for HIV control efforts [2].

Rapid tests for recent infection (RTRIs) are a point-of-care (POC) test developed to be conducted immediately following, or in conjunction with, a new diagnosis of HIV. Based on immunological biomarkers of disease progression, an RTRI and viral load (VL) test are used to complete a recent infection testing algorithm (RITA) to characterize an HIV infection as recent (i.e., likely having acquired HIV within approximately the last 12 months) or long-term [3]. The testing process involves pricking the finger of the client for a drop of blood; results are available in 20 minutes, during which time clients are asked basic demographic and screening questions, (including if they were previously diagnosed with HIV or have received or currently receive antiretroviral therapy (ART)), geographic, and risk factor questions. Dried blood spot (DBS) specimens are additionally collected from clients with an RTRI recent result for VL testing to decrease misclassification among persons not reporting they are already on treatment [4, 5].

In 2019, the Malawi Ministry of Health (MoH) piloted the integration of this novel assay into HIV Testing Services (HTS) in Blantyre District before expanding to further districts. Adding to previously existing HIV systems, recent infection surveillance provides near-real time data to be used by public health decision makers to triangulate with other data sources and guide response to geographic and demographic areas of ongoing transmission. In Malawi, individual results are not returned to clients as results do not affect clinical treatment and are not intended for individual level response, but rather provide a signal of increased transmission [6, 7].

In April 2019, healthcare workers (HCWs) at 23 health facilities in Blantyre district were trained to conduct RTRIs and prepare DBS for VL testing. All HCWs attended a three-day, in-person training followed by a refresher training with role-play at their facility, on the day of site-activation. During both trainings, each HCW practiced conducting RTRIs and documenting results under direct supervision.

By the end of 2021, 24 countries were implementing recent infection surveillance, but no literature was published describing user experiences conducting point-of-care recent infection assays within routine HIV testing services (HTS). Understanding HCW acceptability and feasibility of integration within HTS is important to sustainability, coverage, and generating recommendations for program strengthening. In Malawi, during early implementation, a cross-sectional survey among HCWs explored the acceptability and feasibility of integrating this new surveillance component into routine HTS.

## Methods

In September 2019, following a one-day training, five research assistants (males and females) conducted in-person interviews among HCWs to collect basic demographic information and explore time burden, perceptions, acceptability, and feasibility of recent infection surveillance. A semi-structured survey, with a combination of quantitative and qualitative questions, was used to assess HCWs’ experiences with trainings, supervision, and conducting recent infection testing over the previous 5 months. The study was reviewed in accordance with the U.S. Centers for Disease Control and Prevention (CDC) human research protection procedures and determined to be research, but CDC investigators did not interact with human subjects or have access to identifiable data or specimens for research purposes. The study received ethical approval from Malawi National Health Sciences Research Committee.

Using a tablet-based form in Open Data Kit (ODK) Collect, the research assistants administered an in-person 31-question survey (Additional file 1: Appendix 1). We interviewed a representative convenience sample among the 131 HCWs trained and actively implementing recent infection testing. HCWs were eligible to participate if they were: currently employed at a health facility where recent infection surveillance was initiated, at or before, April 15, 2019; completed at least one RTRI with a patient specimen; and consented to participate in the survey (Additional file 2: Appendix 2). All eligible and present HCWs were recruited for interviews at their respective health facilities from September 5-11, 2019. Research assistants, with experience in conducting interviews, were selected from districts outside Blantyre to mitigate privacy concerns. Informed consent was obtained from all individual participants included in the study. Interviews were conducted in a private location at the health facility in English or Chichewa, the local language, according to the HCW’s preference. Quantitative data were analyzed using STATA version 14.0 and summarized using descriptive statistics. The qualitative portion of the survey explored HCWs' views through open-ended responses. These responses were documented in ODK Collect in the participant’s spoken language and translated if needed from Chichewa to English. As data were being collected, thematic analysis was used to investigate segments of open-ended text that offered insight into user experiences. The emerging themes generated were grouped and used to triangulate and better understand the quantitative information generated [8]. Final themes were summarized and used to explore specific areas of need, and observations made by HCWs.

## Results

A total of 119 HCWs from all 23 health facilities were approached, and all consented to the interview. The median age of participants was 32 years (range: 22-56), and 53% were female. The median years of professional experience was 5 years (range: 1-22) and 116 out of 119 (97%) were health assistants or diagnostic assistants who were specifically trained to conduct HIV diagnostic testing and counseling. Participants worked at multiple health facility departments where HIV testing services are offered, including HTS (47%), outpatient department (10%), antenatal clinic (9%), family planning/gynecology (8%), and inpatient department (8%).

The majority (82%) of HCWs reported the RTRI as ‘easy’ or ‘very easy’ to use. Additionally, 86% of participants reported the RTRI as ‘the same’ or ‘easier’ to use when compared to the routine HIV diagnostic rapid tests. HCWs perceived clients as accepting of recent infection testing, with 100% reporting clients as ‘somewhat’ (18%) or ‘very accepting’ (82%). However, 68% estimated spending ≥20 additional minutes for this activity, beyond routine HTS, for every client newly diagnosed with HIV, with documentation (29%) and DBS preparation (29%) taking the most additional time. The most commonly cited challenges with implementing recent infection testing were the amount of additional time (*n* = 39), clients wanting their individual test results (*n* = 12), and collecting blood by finger prick for the RTRI and/or DBS (*n* = 11). Approximately half of participants (51%) reported RITA requiring, on average, at least two finger pricks in addition to the HIV testing algorithm with each client (Table 1).

**Table 1:** Training and Supervision, Time, Perception and Acceptability of Rapid Testing for Recent Infection for HIV among Health Care Workers in Blantyre District, Malawi, September 2019

***n*** = 119	*n* (%) or median and range
**Training and Supervision**	
** What type of training do you recommend for new colleagues who will be doing recent infection testing? (select all that apply)**	
3- day MoH training	109 (92%)
On-site training by colleagues	24 (20%)
On-site training by recent infection site supervisors	30 (25%)
** How many times have you met with your recent infection site supervisor every month (on average)?**	median: 4 range:1-10
** Which of the following written materials have you used when you perform a recent infection test (select all that apply)?**	
Integrated Algorithm	109 (92%)
Recent Infection SOP	114 (96%)
RTRI Job Aid	102 (86%)
** Have you done a recency test QC exercise since your site started in April?**	
Yes	116 (97%)
No	3 (3%)
** If yes, how many times have you completed a QC since April?**	median: 3 range: 1-20
** From your experience, what are some of the challenges in recent infection testing?**	
Extra time and work	39 (33%)
Not providing results to clients	12 (10%)
Collecting blood via finger prick	11 (9%)
Consent	7 (6%)
Trusting the results	7 (6%)
**Time**	
**How much additional time do you think recent infection testing takes per client?**	
≤ 9 min	21 (18%)
10 - 19 min	18 (15%)
≥ 20 min	80 (67%)
**How much additional time does completing consent for recent infection testing take per client?**	
≤ 9 min	83 (70%)
10 - 19 min	25 (21%)
≥ 20 min	11 (9%)
**What part of recent infection testing takes the most additional time?**	
Consent	19 (16%)
Finger pricks	1 (1%)
Documentation	35 (29%)
DBS	35 (29%)
Getting supplies ready	7 (6%)
Waiting for RTRI results	20 (17%)
**Perceptions and Acceptability**	
**Overall, performing the RTRI is:**	
Somewhat easy or very easy	98 (82%)
Neither difficult nor easy	11 (9%)
Somewhat easy or very difficult	10 (8%)
**In your opinion, are clients accepting of recent infection surveillance?**	
Very accepting	97 (82%)
Somewhat accepting	22 (18%)
Not accepting	0 (0%)
**How does performing the RTRI compare to performing another rapid test?**	
RTRIs are easier	29 (24%)
Same	74 (62%)
RTRIs are harder	16 (14%)
**On average, how many additional finger pricks do you do when you perform a recent infection test (with one client)?**	
No additional fingerpricks, I can get enough blood from the fingerpricks for the HTS algorithm	6 (5%)
1 additional fingerprick	52 (44%)
2 additional fingerpricks	43 (36%)
>2 additional fingerpricks	18 (15%)
**It is important to know if a person was infected with HIV in the last 12 months.**	
Agree	101 (85%)
Neither agree nor disagree	9 (8%)
Disagree	9 (8%)
**People who take the recent infection test should know the outcome of it.**	
Agree	41 (35%)
Neither agree nor disagree	27 (23%)
Disagree	51 (43%)
**It is easy to enter the results of the test and client information quickly in the recency register.**	
Agree	108 (91%)
Neither agree nor disagree	3 (3%)
Disagree	8 (7%)
**The use of the recency test in HTS improves the services offered to the clients.**	
Agree	45 (38%)
Neither agree nor disagree	37 (31%)
Disagree	37 (31%)

*Abbreviations*: *MoH* Ministry of Health, *SOP* Standard Operating Procedures, *RTRI* Rapid Test for Recent Infection, *QC* Quality Control

Qualitative exploration of additional training needs identified that DBS preparation and the informed consent process were most frequently cited areas of need for further training. Some participants cited instances of not knowing how to respond to clients’ request for recent infection test results and being unclear on consenting procedures for those with diminished decision-making capacity. When asked what type of trainings they recommend for new colleagues, 92% of participants recommended the in-person three-day training format and 25% recommended on-site training by surveillance supervisors at the time of facility activation.

The median times HCWs reported meeting with recent infection surveillance supervisors was four times per month (range:1-10). Ninety-eight percent of participants reported completing at least one round of standard quality control specimen testing using RTRIs, with an average of performing three rounds since implementing 5 months prior. HCWs reported using printed guidance materials when performing recent infection testing, such as the job aids (86%), the testing algorithm graphic (92%), and the standard operating procedures (96%). The reliance on these documents was reflected in open-ended responses communicating the need for updated materials and information to fill knowledge gaps and provide facility-specific clarifications.

Many HCWs (85%) reported understanding the importance of knowing if someone was recently infected with HIV to guide future MoH HIV prevention and testing activities. However, the idea of providing clients with their RTRI results elicited conflicting responses from the HCWs. Participants who stated clients should receive their results (35%) believed that knowing the result may enhance partner notification services or the client’s willingness to accept their HIV diagnosis, or that clients had the right to know their test results. HCWs that did not believe clients should receive their results (43%) had concerns that providing recent infection test results may instigate chaos, confusion, or violence among partners and families. One HCW described the surveillance activity as *“strictly for the government to know to come up with further intervention,”* explaining that “*even if the clients know, they won’t benefit anything.”* Another HCW went further and described how a recent infection test result may negatively impact a client, *“it would tear families apart, especially in cases of a couple whereby one’s results show she or he has been recently infected.”* On the other side, one HCW said if they received their results, *“they would plan for their future better and it would also encourage behavior change,”* while several believed *"it’s the client’s right to know their results."*

When conducting recent infection testing, HCWs had reservations related to difficulty in responding to clients’ questions around why individual-level test results were not returned (*n* = 95), unclear direct benefit of the surveillance activity to the client (*n* = 38), and lack of financial incentives for additional work for HCWs and clients (*n* = 21). Some also noted a need for further training on how to collect and prepare DBS cards (*n* = 11) and wanting to be informed of the planned public health response using the test results (*n* = 9).

## Discussion

This study highlights the perspectives of HCWs in implementing a novel assay and is the first study to assess experiences of integrating recent infection surveillance using RTRIs into a country’s national HIV testing program. The survey findings are consistent with the limited existing literature, demonstrating high feasibility and acceptability among HCWs [9, 10]. RTRIs were perceived to be easy-to-use and similar in difficulty to conducting a rapid HIV diagnostic test. Other publications have described the potential importance of integrating this surveillance into HTS as part of a multi-pronged approach to guide prevention activities, monitor trends, and assess the efficacy of programmatic or treatment interventions [11]. Findings from another study in Kenya and Zimbabwe highlighted the importance of incorporating HTS information to accurately classify recent infections and suggested that, when rolled-out nationally, recent infection surveillance can further guide primary prevention efforts [12].

Most HCWs appreciated the added value in recent infection surveillance, explaining how the results could be used by MoH and partners to guide public health response. Likewise, HCWs reported perceived high acceptability among clients. Consistent facility-level support for the new activity through supervision visits was appreciated by most HCWs. Overall, HCWs found the sample collection process easy to conduct and trainings to be beneficial. The three-day in-person trainings were viewed by HCWs as the best method to prepare them to conduct HIV recent infection testing.

While the majority reported ease of use of the RTRI, HCWs also identified challenges with implementation within routine HTS, including time burden and the need for at least two additional finger pricks to complete the testing algorithm with an RTRI recent result. The additional time burden may not represent a significant time loss at health facilities with few new HIV diagnoses; however, burden may increase with the assessment of additional behavioral and risk factors or a high number of new HIV diagnoses at high-volume or understaffed facilities and entry points. Voluntary assisted partner notification (VAPN) services were initiated, as a study, at high-volume facilities in Blantyre the same year as recent infection testing, increasing HCW responsibilities for every newly diagnosed client in addition to recent infection testing [13]; VAPN has since become national policy in Malawi. A more in-depth assessment of the time burden for both HCWs and clients may be warranted at facilities implementing multiple HTS activities.

The number of additional finger pricks needed to complete testing if RTRI results are recent may be considered excessive by the clients. Additional training on finger prick procedures and bundling tests to minimize the need for repeated pricks, may be helpful. A study by Thakar et al. reported on clients preferring venous blood samples over finger pricks because of discomfort of multiple pricks if sufficient volume is not obtained the first time [14]. If blood is drawn for baseline labs prior to antiretroviral therapy (ART) initiation, repositioning recent infection testing to be conducted within ART initiation procedures may be a consideration. However, this approach may result in loss of surveillance data from persons who are newly diagnosed with HIV but do not initiate ART for various reasons.

Among the participant responses, there was not a consensus among HCWs as to whether clients should receive their individual recent infection test results. In situations where recent infection results are provided to clients, potential adverse events such as intimate partner violence, discrimination, and criminalization have been cited as concerns and, this has lead to varying approaches in returning results across countries [6, 9, 10, 15]. HCWs should be trained to navigate client discussions regarding interpretation of test results and prevent potentially harmful situations previously described. Research is onging to ascertain potential benefits, harms, or negative repercussions of result provision to clients. In countries where recent infection test results are not provided to clients, more comprehensive scripts may assist HCWs in explaining to clients why they are not provided their RTRI result.

HCWs request for facility-level summary results may reflect the overall importance of two-way communication as a contributor to job satisfaction and job performance in non–healthcare and healthcare settings alike [16–18]. Other studies have shown that HCWs found encouragement in community appreciation and perceived government and partner support, which can be provided through regular dissemination of regional- or facility-level findings from the MoH [16, 19]. The use of aggregated surveillance results to inform public health response activities may also reinforce the importance of recent infection surveillance among HCWs and gain support for sustainability.

The survey also identified topics to prioritize for refresher trainings and supportive supervision, including DBS and informed consent, both of which are part of the current mandatory in-person three-day training. The majority of the HCWs who participated in the survey reported using printed materials to perform recent infection testing. Up-to-date job-aids, standard operating procedures, and guidelines readily available at the facility, and continued supervision will reinforce previous trainings.

Limitations in this study include social desirability bias, which we tried to address in selecting independent research assistants from outside of Blantyre district (who were not part of recent infection trainings or supervision), as well as encouraging the interviews to take place in a private location and in the participant's preferred language. All HCWs present were invited to participate to prevent sampling bias, and while all HCWs approached consented to the survey, not all eligible HCWs were present during the scheduled interview day. Responses may not represent all those trained, but those consented do represent 91% (119/131) of all HCWs performing recent infection testing at the time. The survey was designed to capture the attitudes and experiences of HCWs, thus their responses may not accurately capture the experiences of clients. Further research is needed to understand clients’ experiences. Finally, this survey was conducted among a small sample of HCWs in one district in Malawi and may not be generalizable to other settings. Although similarities do exist among countries implementing, their trainings and integration of recent infection surveillance into HTS vary. As of September 2021, Malawi was one of 24 countries implementing recent infection surveillance. More than half of the countries implementing recent infection surveillance utilized HCWs to integrate the RTRI at HTS and did not provide individual test results. We therefore believe our findings may be generalizable to other countries.

## Conclusion

Overall, HCWs perceived RTRIs to be acceptable, easy-to-use, and valuable. The feasibility and acceptability of recent infection testing by HCWs is important to the success of this program and ultimately the quality of the surveillance data to effectively guide prevention and testing activities. As data collection continues, future analyses, such as recent HIV infection surveillance summaries and hotspot detection, will be shared with HCWs so they can see how the data they collect are useful in guiding public health response plans. Sustaining a skilled, motivated, and well-supported workforce to continue recent infection testing will require effort on several levels, including integration of services into the national framework for HIV/AIDS. Acceptability and feasibility of HIV recent infection surveillance by HCWs is essential for the long-term collection of accurate data which will contribute to the overall success in ending the HIV epidemic.

## Supplementary Information

**Additional file 1.**

**Additional file 2.**

## Data Availability

The dataset supporting the conclusions of this article is available through contacting the corresponding author.

## Abbreviations

AIDS: Acquired immunodeficiency syndrome
ART: Antiretroviral treatment
CDC: Centers for Disease Control and Prevention
DBS: Dried blood spots
HIV: Human immunodeficiency virus
HCW: Healthcare worker
HTS: HIV testing services
MoH: Ministry of Health
ODK: Open Data Kit
RTRI: Rapid test for recent infection
RITA: Recent infection testing algorithm
VAPN: Voluntary assisted partner notification
VL: Viral load
