# Correction: Acceptability and feasibility of HIV recent infection surveillance by healthcare workers using a rapid test for recent infection at HIV testing sites — Malawi, 2019

**DOI:** 10.1186/s12913-022-08303-9

**Published:** 2022-07-15

**Authors:** Melissa M. Arons, Kathryn G. Curran, Malango Msukwa, Joe Theu, Gabrielle O’Malley, Alexandra Ernst, Ireen Namakhoma, George Bello, Carson Telford, Vedapuri Shanmugam, Bharat Parekh, Evelyn Kim, Trudy Dobbs, Danielle Payne, Salem Gugsa

**Affiliations:** 1 Division of Global HIV and Tuberculosis, Center for Global Health, Centers for Disease Control and Prevention, Atlanta, GA USA; 2 Department of Global Health, International Training and Education Center for Health (I-TECH), University of Washington, Lilongwe, Malawi; 3 Department of Global Health, International Training and Education Center for Health (I-TECH), University of Washington, Seattle, WA USA; 4 Global Strategic Information, Institute for Global Health Sciences, University of California, San Francisco, CA USA; 5 Department of HIV AIDS, Ministry of Health, Lilongwe, Malawi; 6 Division of Global HIV and Tuberculosis, Center for Global Health, Centers for Disease Control and Prevention, Lilongwe, Malawi

**Correction: BMC Health Serv Res 22, 341 (2022)**

**https://doi.org/10.1186/s12913-022-07600-7**

Following publication of the original article [1], the authors identified an error in the affiliations stated for the last author, Salem Gugsa. At the time of the manuscript development, the author’s affiliation was assigned to 3 instead of 4.

The affiliations assignment is corrected in the author list of this Correction article and the original article [1] has been updated.
