# Supplementary material for: Acceptability and feasibility of HIV recent infection surveillance by healthcare workers using a rapid test for recent infection at HIV testing sites — Malawi, 2019
Source: BMC Health Serv Res. 2022 Mar 15;22:341. doi: 10.1186/s12913-022-07600-7 (PMC8922771; doi:10.1186/s12913-022-07600-7)
Supplement: Supplementary file 1 — Additional file 1. [file 12913_2022_7600_MOESM1_ESM.docx]

***Appendix 1***

**RECENT INFECTION SURVEILLANCE PHASE 1 EVALUATION INTERVIEW GUIDE**

**Appropriateness, acceptability and feasibility of integrated rapid test for recent infection (RTRI) and evaluation of Sedia Asante and Maxim Swift**

**Objectives:** To explore health workers’ experiences implementing point of care testing for recent infection into HTS, including their perception of the test and additional time spent on the activity.

Introduce the purpose of the study – its aims and scope:

- Assure participant of confidentiality and how it will be maintained
- Ask for their consent to participate (fill in consent form)
- If they do not consent, then do not complete the interview with them. If they do consent, remind them the Interview will take approximately 20 minutes, and they can stop the interview, opt-out or interrupt with questions or clarification at any point during the interview.

Health care worker survey inclusion criteria:

- Employed at a health facility where recent infection surveillance was initiated at or before April 15, 2019 and recent infection testing is currently being performed
- Has completed at least one RTRI
- Giving voluntary informed consent for survey

Exclusion criteria:

- Does not consent
- Has not completed an RTRI

| **Interviewer ID** |  | **Clinic Name** |  |
| --- | --- | --- | --- |
| **Date of Interview** |  | **HTS Entry Point** |  |
| **Survey ID** |  | **What RTRI is in use?**  *(circle one)* | Maxim Swift,  Sedia Asante |
| **Interviewee Consented** |  |  |  |

| **General Recent Infection Questions** | | |
| --- | --- | --- |
|  | Approximately ***how many clients do you test*** with the RTRI in **one week**? | _________ approximate number of clients tested in one week |
|  | In your opinion, are clients accepting of recent infection surveillance? | 1. Very accepting 2. Somewhat accepting 3. Not accepting |
|  | Overall, performing the RTRI is: | 1. Very Difficult 2. Somewhat Difficult 3. Neither Difficult nor Easy 4. Somewhat Easy 5. Very Easy |
|  | Using the Swift (Maxim) pipette/Asante loop for the blood sample is: | 1. Very Difficult 2. Somewhat Difficult 3. Neither Difficult nor Easy 4. Somewhat Easy 5. Very Easy |
|  | Reading the lines on the RTRI is (For example, reading the three lines on the test device to record the results.): | 1. Very Difficult 2. Somewhat Difficult 3. Neither Difficult nor Easy 4. Somewhat Easy 5. Very Easy |
|  | How does performing Swift (Maxim)/Asante compare to performing an HIV rapid test? | Recent infection test is easier  Same   1. Recent infection test is harder |
| **Training Supervision and Guidance** | | |
|  | Did you receive training on recent infection testing? | Yes  No |
|  | If yes, what was the training? (select all that apply) | 1. 3-day MOH training 2. On-site training by colleagues 3. On-site training by Recent Infection Site Supervisors 4. Other |
|  | If other, please describe: | Comments: |
|  | Are there any skills/subjects you would have liked more training on before starting recent infection testing? | Yes  No |
|  | b.If yes, please describe: | Comments: |
|  | What type of training do you recommend for new colleagues who will be doing recent infection testing? (select all that apply)? | 1. 3-day MOH training 2. On-site training by colleagues 3. On-site training by Recent Infection Site Supervisors 4. I don’t know 5. Other |
|  | f.If other, please describe: | Comments: |
|  | How many times do you meet with the Recent Infection Site Supervisors per month? | ________ times per month |
|  | Which of the following written materials have you used when you perform a recent infection test (select all that apply)? | (see below) |
|  | 1. Integrated algorithm | Yes  No |
|  | 1. Recent Infection SOP | Yes  No |
|  | 1. RTRI Job Aid | Yes  No |
|  | 1. Other, please describe: | 1. Comments: |
|  | 1. none of the above |  |
|  | Have you done a recency test QC exercise since your site started with TRACE in April?  (performing the RTRI on the 3 QC specimens with the Recent Infection Site Coordinator) | Yes  No |
|  | b. If yes, how many times have you completed a QC since April? (including site activation) | ______________times performed QC |
|  | On average, how many additional finger pricks do you do when you perform a recent infection test (with one client)?: | 1. No additional fingerpricks, I can get enough blood from the fingerpricks for the HTS algorithm 2. 1 additional fingerprick 3. 2 additional fingerpricks 4. > 2 additional fingerpricks |
|  | True or False? Sometimes I am not able to collect DBS with recent infection testing. | True  False |
|  | b. Why is DBS difficult to collect? | Comments: |
| **Time** | | |
|  | How much additional time do you think recent infection testing takes per client? | 1. Less than 5 minutes 2. 5 to 9 minutes 3. 10 to 19 minutes 4. 20 to 29 minutes 5. 30 minutes or more |
|  | How much additional time does completing consent for recent infection testing take per client? | 1. Less than 5 minutes 2. 5 to 9 minutes 3. 10 to 19 minutes 4. 20 to 29 minutes 5. 30 minutes or more |
|  | What part of recent infection testing takes the most additional time? | 1. Consent 2. Fingerpricks 3. Documentation & Paperwork 4. DBS 5. Getting the supplies ready 6. Other |
|  | b. If other, please describe | Comments: |
|  | What questions do clients have about the recent infection test? | Comments: |
|  | If you remember being trained on the other POC test for recent infection, do you have a preference for which test to use at your clinic? | 1. Swift (Maxim) 2. Asante 3. Don’t remember 4. No preference 5. Trained on only 1 POC test |
|  | b. If you have a preference, why do you prefer this test? | Comments: |
|  | From your experience, what are some of the challenges in recent infection testing? | Comments: |
| **Attitudes towards Recent Infection Testing** | | |
|  | It is important to know if a person was infected with HIV in the last 12 months. | Agree  Neither Agree or Disagree  Disagree |
|  | b.If agree or disagree, why do you feel this way? | Comments: |
|  | People who take the test for recent infection should know the outcome of it. | Agree  Neither Agree or Disagree  Disagree |
|  | b.If agree or disagree, why do you feel this way? | Comments: |
|  | The use of the recency test in HTS improves the services offered to the clients. | Agree  Neither Agree or Disagree  Disagree |
|  | b.If agree or disagree,why do you feel this way? | Comments |
|  | It is easy to enter the results of the test and client information quickly in the recency register. | Agree  Neither Agree or Disagree  Disagree |
|  | Do you think improvements should be made to the recent infection register to make it more user friendly? | Yes  No |
|  | b. If yes, how would you improve the register to make it more user-friendly? | Comments: |
| **Background of Interviewee** | | |
|  | Gender | 1. Male 2. Female 3. Other |
|  | Age | ________years |
|  | What is your job title? | 1. Health Diagnostic Assistant (HDA) 2. Health Surveillance Assistant (HSA) 3. Nurse 4. Clinical Officer 5. Medical Assistant 6. Lab Tech 7. Site Supervisor 8. Other |
|  | b. Other | Comments: |
|  | How many ***years*** have you been providing HIV testing services? | _________ years |
