# Supplementary material for: Acceptability and feasibility of HIV recent infection surveillance by healthcare workers using a rapid test for recent infection at HIV testing sites — Malawi, 2019
Source: BMC Health Serv Res. 2022 Mar 15;22:341. doi: 10.1186/s12913-022-07600-7 (PMC8922771; doi:10.1186/s12913-022-07600-7)
Supplement: Supplementary file 2 — Additional file 2. [file 12913_2022_7600_MOESM2_ESM.docx]

# *Appendix 2*

# Recent Infection Surveillance Phase 1 Evaluation Consent form

**Consent Form – Health Care Workers Interview Introduction**

You are being asked to take part in an evaluation being carried out by the International Training and Education Center for Health (I-TECH), the US Centers for Disease Control and Prevention (CDC) Malawi, University of California San Francisco, and Malawi Ministry of Health to explore health workers’ experiences implementing rapid tests for recent infection (RTRI). This consent form explains what will happen in the interview, any risks and benefits, and how we will protect your private information.

**Purpose of the Study and Study Requirements**

**What is the Evaluation?** We are doing face-to-face interviews with health care workers at 23 clinics implementing RTRIs in Blantyre District. The interview will take about 15 minutes and include questions on: timeliness, acceptability, training and supervision, and ease of integrating recent infection testing. While names will not be collected, age, sex, clinic name and HTS entry point will be documented. Every effort to keep data secure will be made and results by site will not be shared. The information will help the MOH in supporting recent infection surveillance and making programmatic, training and supply decisions.

**What is the purpose of this study?** New rapid tests for recent infection (RTRI), (e.g., Sedia Asanté and Maxim Swift) are being used in Blantyre at health facilities providing HTS services since March, 2019. We will interview HTS providers that participate in and support HIV testing to evaluate the appropriateness, acceptability and feasibility of integrating recent infection surveillance using RTRIs.

**Why have I been invited to take part?** You are being asked to take part in this evaluation because we would like to know what you and your colleagues think about recent infection surveillance.

**What will happen if I take part?** If you agree to take part, you will be asked questions for approximately 15 minutes The interview will be guided by myself. I will take notes and ask you both multiple choice questions and open ended questions. We will ask about experiences with, and attitudes towards recent infection testing and support around this service.

**What are the risks of the interview?** We do not expect any risks to you for being in this study. Your responses will not affect your job or working relations. You do not have to answer any questions that make you uncomfortable. There is a risk that people outside of the study may find out what you said. However, we will do our best to make sure that this does not happen. We will not include your name on any of the reports, so that no-one will know your information specifically. We will only share the combined results and will not report back to your supervisor or colleagues what you say.

**What are the benefits of participating?** There are no benefits to the interview. However, attending these discussion groups may give you a chance to ask questions and discuss your feelings and concerns. You will also be able to share what you know and your experiences as. Also, the findings from the study will benefit recent infection surveillance in Malawi and other countries.

**Will my being in the study be kept private?** The evaluation team will do its best to protect your privacy and all the information that you provide. Your name will not be included in reports from this study. The information you give us will be stored in a computer that only the study team can access.

**What are my rights as a research participant?** Your participation in this interview is entirely voluntary. If you agree to join, you can refuse to answer any questions you do not want to answer and can stop the interview at any time. Your decision not to answer certain questions or not participate in the interview, will not affect your work or position.

**What will I receive for participating?** You will not receive any money for taking part in the interview.

**What if I have a question or there is a problem?** You may ask any questions about this survey or this consent form now or in the future. If you have any concerns about the study or any complaint about the way you have been treated, please contact:

**Name:** Dr. George Bello

**Organization:** International Training and Education Center for Health (I-TECH)

**Address:** Ministry of Health, PO Box 30377, Lilongwe, Malawi

**Email:** [gafbello@yahoo.co.uk](mailto:gafbello@yahoo.co.uk) **Tel:** +265888892212

If you have any questions about your rights as a participant in this activity, you can contact:

**Name:** Dr. Damson Kathyola

**Address:** Ministry of Health, National Health Sciences Research Committee, P.O Box 30337, Lilongwe, Malawi

**Email:** [dkathyola@gmail.com](mailto:dkathyola@gmail.com) **Phone:** +2651726422/418

**PARTICIPANT:** I have read the information in this consent form including risks and benefits, or it has been explained to me. I have been giving the chance to ask questions about the study. By signing below I agree to take part in the study.

____________________________________________________________________________

(Participant Name) (Signature) (Date)

____________________________________________________________________________

(Researcher Name) (Signature) (Date)

YOU WILL BE GIVEN A COPY OF THIS CONSENT FORM TO KEEP.

**Please document the unique interview ID number before completing the survey**

Interview Unique ID Number:___________________
